# Evaluation of the Liver Toxicity of *Pterocephalus hookeri* Extract via Triggering Necrosis

**DOI:** 10.3390/toxins11030142

**Published:** 2019-03-02

**Authors:** Rui Wang, Zhaoyue Dong, Xiaolong Zhang, Jingxin Mao, Fancheng Meng, Xiaozhong Lan, Zhihua Liao, Min Chen

**Affiliations:** 1 College of Pharmaceutical Sciences, Key Laboratory of Luminescent and Real-Time Analytical Chemistry (Southwest University), Ministry of Education, Southwest University, Chongqing 400715, China; liuchishou@email.swu.edu.cn (R.W.); dzy2018@email.swu.edu.cn (Z.D.); zxlwjw12@email.swu.edu.cn (X.Z.); maomao1985@email.swu.edu.cn (J.M.); mengfcswu@swu.edu.cn (F.M.); 2 TAAHC-SWU Medicinal Plant R&D Center, XiZang Agriculture and Animal Husbandry College, Nyingchi, Tibet 860000, China; lanxiaozhong@163.com; 3 School of Life Sciences, Southwest University, Chongqing 400715, China; zhliao@swu.edu.cn

**Keywords:** *Pterocephalus hookeri*, liver toxicity, n-butanol extract, necrosis, inflammation

## Abstract

*Pterocephalus hookeri* (C. B. Clarke) Höeck, recorded in the Chinese Pharmacopoeia (2015 version) as a Tibetan medicine for the treatment of various diseases, especially rheumatoid arthritis, was believed to possess a slight toxicity. However, hardly any research has been carried out about it. The present study aimed to evaluate the toxicity in vivo and in vitro. Toxicity was observed by the evaluation of mice weight loss and histopathological changes in the liver. Then, the comparison research between ethyl acetate extract (EAE) and n-butanol extract (BUE) suggested that liver toxicity was mainly induced by BUE. The mechanical study suggested that BUE-induced liver toxicity was closely associated with necrosis detected by MTT and propidium iodide (PI) staining, via releasing lactate dehydrogenase (LDH), reducing the fluidity, and increasing the permeability of the cell membrane. Western blot analysis confirmed that the necrosis occurred molecularly by the up-regulation of receptor-interacting protein kinase 1 (RIP1) and receptor-interacting protein kinase 3 (RIP3), as well as the activation of the nuclear factor-kappa-gene binding (NF-κB) signaling pathway in vivo and in vitro. This finding indicated that the liver toxicity induced by BUE from *P. hookeri* was mainly caused by necrosis, which provides an important theoretical support for further evaluation of the safety of this folk medicine.

## 1. Introduction

There is a long history of using traditional Chinese medicine for the treatment of various diseases. It has been recorded that 943 species of toxic plants exist in China, of which more than 500 species are medicinal plants. Moreover, 83 toxic traditional Chinese medicines are included in the Chinese Pharmacopoeia (2015 version). However, only 13 of them have been established using analytical methods [1]. Furthermore, the severe side effects of toxic constituents from herbs, such as aristolochic acid [2], have aroused people’s widespread attention. It is urgent to evaluate toxicities, allowing for a more rational assessment of risks in the clinic application of these medicines. As the main organ of drug metabolism in the body, the liver is more likely to be a target organ damaged by drugs. Studies have shown that herbs were the second cause of drug-induced liver injury in the United States [3]. Additionally, the inappropriate use of herbs may easily induce liver injury, especially in those patients with existing liver diseases [4].

*Pterocephalus hookeri* (C. B. Clarke) Höeck was a widely used Tibetan medicine for the treatment of rheumatoid arthritis (RA), the medicinal part being the entire leaves. It has been recorded to have an anti-inflammatory activity for ethanol and aqueous extracts [5]. As a typical chronic disease, RA needs long-term medication to relieve inflammation and pain, requiring a better understanding of medication guidance and drug safety. Therefore, we aimed to evaluate the liver toxicity of extracts from *P. hookeri* (PH) in vivo and in vitro in this study, and further explore the possible toxicological mechanism.

## 2. Results

### 2.1. Toxic Effects of PH in Mice

**Figure 1:**
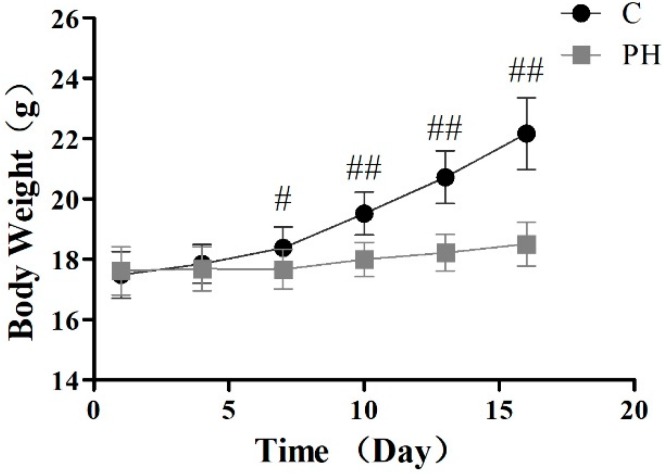
Effects on body weight in mice treated with PH (5 g/kg) during 16 days. Values are expressed as mean ± SD (*n* = 8), # *p* < 0.05, ## *p* < 0.01 compared with the control group.

**Figure 2:**
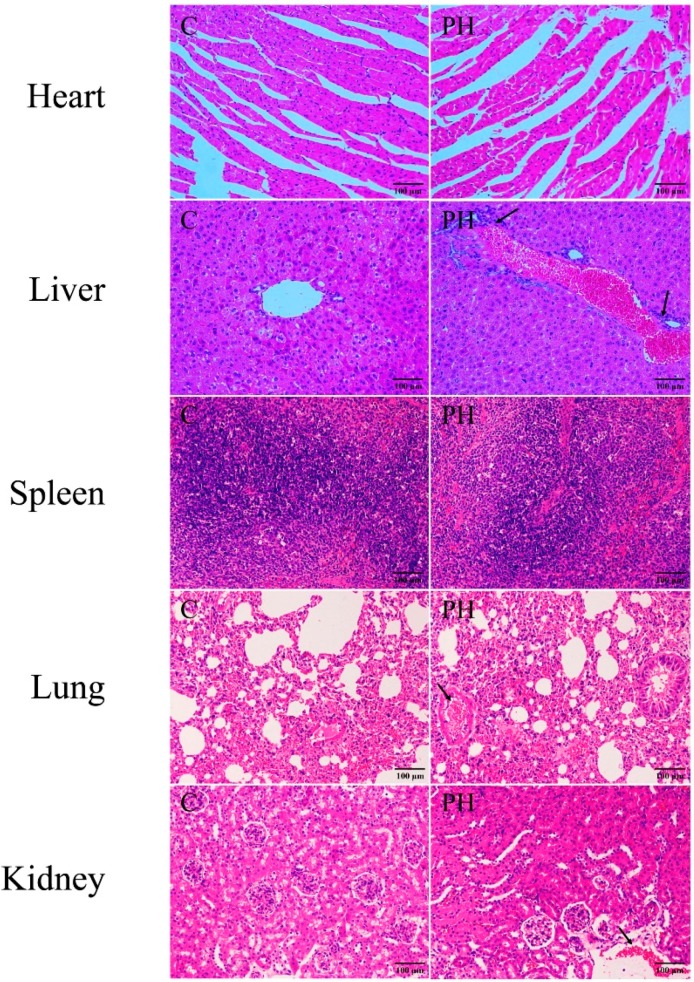
Histopathological analysis of heart, liver, spleen, lung, and kidney treated with PH (hematoxylin and eosin (H&E); ×200).

Body weight is the most intuitive index to reflect health and is used in many research studies [6]. To evaluate the preliminary toxicity of PH toward mice, body weight was monitored every three days until the 16th day. As illustrated in Figure 1, compared with the control group, there was a progressive decrease in weight in the PH group, indicating treatment of 5 g/kg of PH might cause discomfort for mice. Then, the main splanchnic tissues of heart, liver, spleen, lung, and kidney were evaluated for histological examination by hematoxylin and eosin (H&E) staining (Figure 2). The result suggested that the liver section clearly showed inflammation and necrosis in the PH group, compared with the control group. At the same time, less uncertain damage was found in the kidney and lung tissues.

### 2.2. Liver Toxicity of EAE and BUE on Carrageenan-Induced Paw Edema in Mice

**Figure 3:**
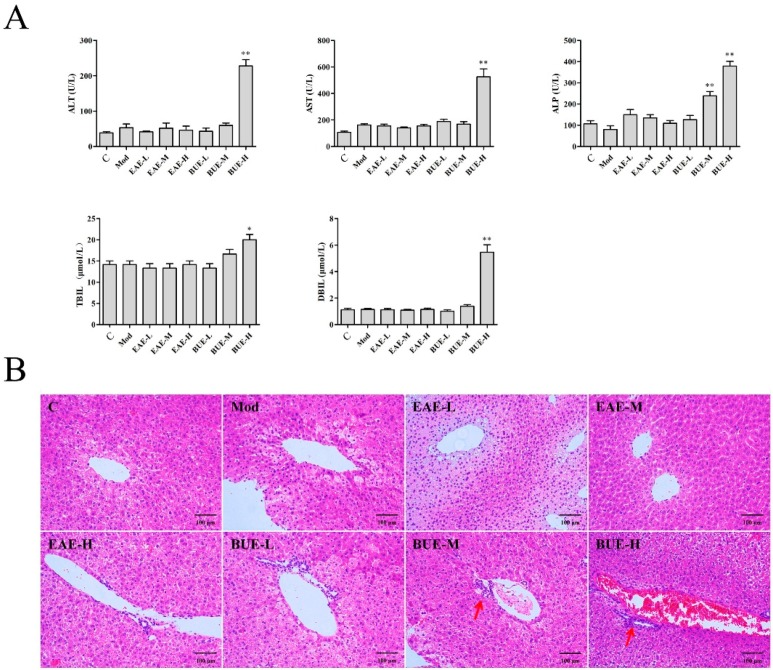
Effects of EAE and BUE on the serum levels of ALT, AST, ALP, TBIL and DBIL (**A**), and histopathological changes (**B**) in the liver. Data are represented as mean ± SD (*n* = 8), * *p* < 0.05 and ** *p* < 0.01 compared with the control group (H&E; ×200).

Petroleum ether extract (PEE), ethyl acetate extract (EAE), and n-butanol extract (BUE) for *P. hookeri* were applied for anti-inflammatory evaluation in mice. The low (L), middle (M) and high (H) dose of PEE, EAE and BUE were set as 200, 400, 800 mg/kg. The result showed that both EAE and BUE significantly reduced the carrageenan-induced paw edema. However, PEE did not exhibit obvious anti-inflammatory results in this research (Table S1). For liver biochemical indicator detection, the BUE-H group significantly increased serum alanine aminotransferase (ALT), aspartate transaminase (AST), alkaline phosphatase (ALP), direct bilirubin (DBIL), and total bilirubin (TBIL) in mice, all of which indicated liver injury. However, the EAE groups showed no obvious influences for these compared with the control group (Figure 3A). Liver sections were stained with H&E to intuitively evaluate histological changes in the liver (Figure 3B). Inflammatory cell infiltration and vascular congestion were observed in the BUE-H group, suggesting necrosis occurred. EAE groups showed no liver toxicity, exhibiting normal cellular structure.

### 2.3. Effects of EAE and BUE on the Expression of NF-κB Signaling Pathway In Vivo

**Figure 4:**
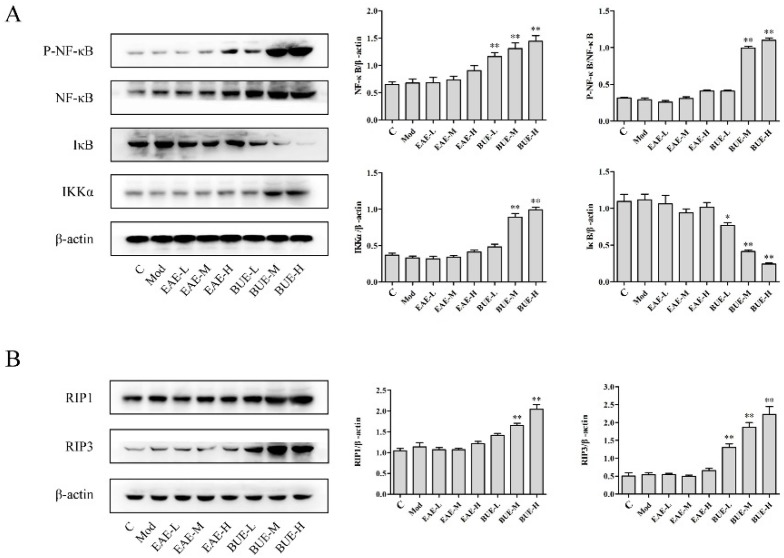
Effects of EAE and BUE on (**A**) NF-κB signaling pathway and (**B**) RIP1 and RIP3 for liver tissues in carrageenan-induced mice. Results are expressed as mean ± SD (*n* = 3), * *p* < 0.05, ** *p* < 0.01 compared with the control group.

Protein expression in classic inflammatory nuclear factor-kappa-gene binding (NF-κB) signaling pathway in liver was detected. Phospho-NF-κB (P-NF-κB), NF-κB, and inhibitor of NF-κB kinase α (IKKα) were significantly increased in BUE groups in comparison with the control group (Figure 4A). In contrast, inhibitor of NF-κB (IκB) level decreased in BUE groups compared with the control group (*p* < 0.01). However, there was no significant change in EAE groups.

### 2.4. Effects of EAE and BUE on the Expression of Necrosis-Related Proteins In Vivo

Cell necrosis caused by exogenous drugs is one of the important causes of liver injury. Thus, we also examined the expression of receptor-interacting protein kinase 1 (RIP1) [7] and receptor-interacting protein kinase 3 (RIP3) [8], which were the two key proteins that induced activation of necrosis signal. The treatment of BUE induced the stimulatory effects of necrosis on RIP1 and RIP3 levels (*p* < 0.01, Figure 4B). EAE groups showed no significant regulation of the expression of RIP1 and RIP3. This result was analogous to morphological observation in the liver section.

### 2.5. Effect of EAE and BUE on L-02, RAW264.7, and HUVECs

**Figure 5:**
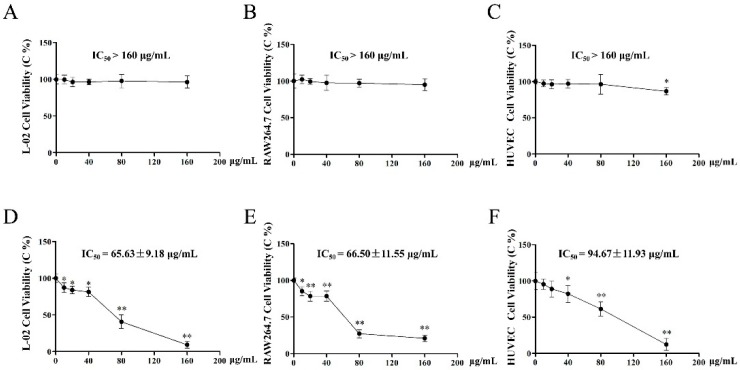
Effects of (**A–C**) EAE and (**D–F**) BUE on viabilities of L-02, RAW264.7, and HUVEC cells. Results are expressed as a percentage of the control group, which was set at 100%; values are expressed as means ± SD (*n* = 5), * *p* < 0.05, ** *p* < 0.01 compared with the control group.

L-02, RAW264.7, and HUVECs were selected for in vitro toxicity research. As a result, even with the treatment of EAE at the highest dosage (160 μg/mL), these three cells exhibited a slight proliferation inhibitory effect with the IC_50_ > 160 μg/mL (Figure 5A–C). However, cell viabilities in BUE groups were decreased significantly in a dose-dependent manner, especially at 160 μg/mL, with the IC_50_ at 65.63 ± 9.18, 66.50 ± 11.55, and 94.67 ± 11.93 μg/mL, respectively (Figure 5D–F).

**Figure 6:**
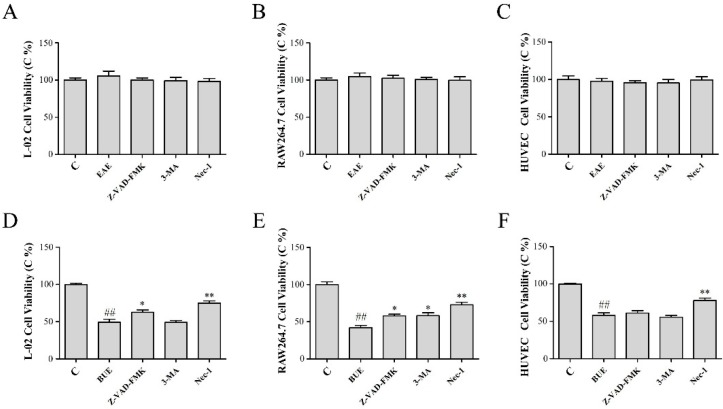
Effects of (**A–C**) EAE and (**D–F**) BUE on viabilities in L-02, RAW264.7, and HUVEC cells treated with Z-VAD-FMK, 3-MA, and Nec-1. Results are expressed as a percentage of the control group, which was set at 100%; values are expressed as means ± SD (*n* = 5), ## *p* < 0.01 compared with the control group, * *p* < 0.05, ** *p* < 0.01 compared with the BUE group.

In order to further explore the main cause affecting of cell viability, Z-VAD-FMK [9], 3-methyladenine (3-MA) [10], and necrostatin-1 (Nec-1) [11] were used to interfere with cell apoptosis, autophagy, and necrosis, respectively. The results (Figure 6A–C) showed that in EAE (80 μg/mL) groups, there was no significant influence on the viability of the three cells with the various added inhibitors. Furthermore, the treatment of Z-VAD-FMK and 3-MA exhibited weak changes in the viability of these three cells in BUE (80 μg/mL) groups (Figure 6D,E). However, the treatment of Nec-1 could reverse significantly (*p* < 0.01) the reduction of cell viability induced by BUE, indicating that the toxicity of BUE was closely related to cell necrosis.

**Figure 7:**
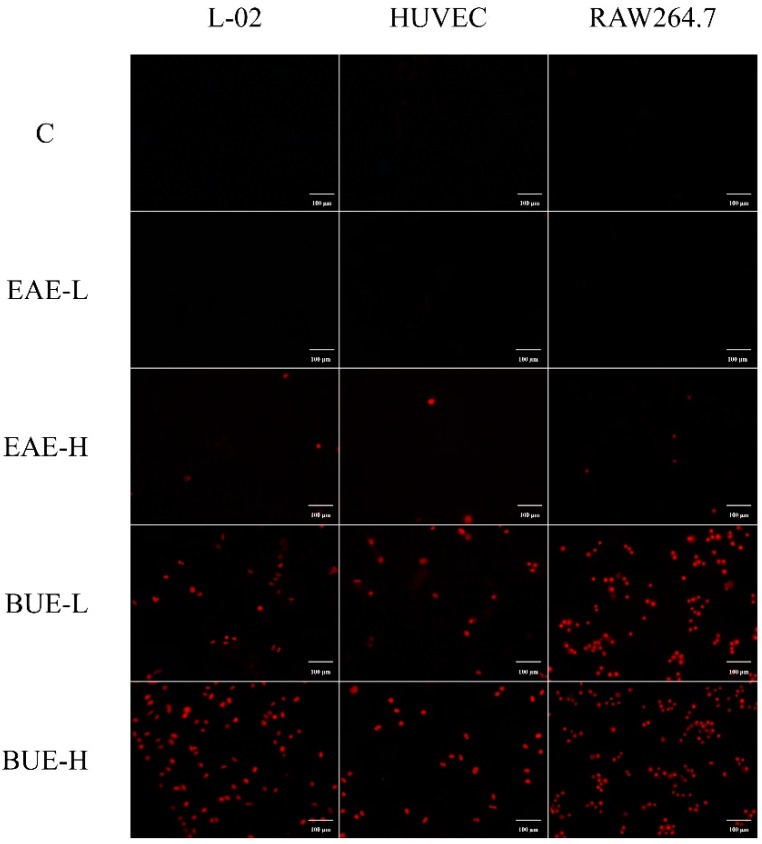
Necrotic effects induced by EAE and BUE in L-02, RAW264.7, and HUVEC cells by PI staining (×200).

Propidium iodide (PI) staining was commonly applied to identify necrotic cells. As shown in Figure 7, EAE groups presented weak red fluorescence in 80 and 160 μg/mL. In contrast, red fluorescence in BUE groups at 80 and 160 μg/mL was significantly enhanced compared with the control group, especially in the BUE-H group. Therefore, the necrosis was intuitively confirmed by obvious red fluorescence.

### 2.6. Effects of EAE and BUE on NF-κB Signaling Pathway In Vitro

**Figure 8:**
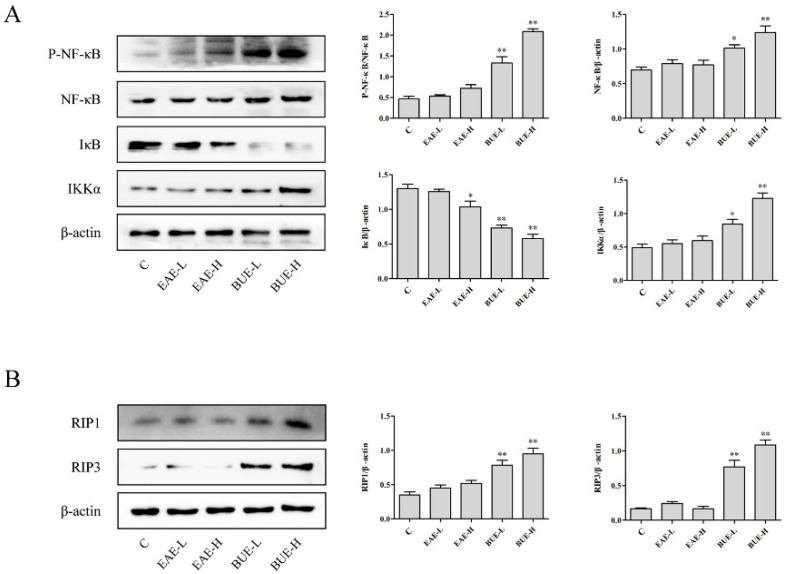
Effects of EAE and BUE on (**A**) NF-κB signaling pathway and (**B**) RIP1 and RIP3 in L-02 cells. Results are expressed as mean ± SD (*n* = 3), * *p* < 0.05, ** *p* < 0.01 compared with the control group.

Western blot results in L-02 cells were similar with those in mice liver tissues. As shown in Figure 8A, the protein level of P-NF-κB, NF-κB, and IKKα increased significantly in BUE groups, whereas the level of IκB significantly decreased, in comparison with the control group (*p* < 0.01). In addition, EAE groups exhibited no significant effect on NF-κB signaling pathway.

### 2.7. Effects of EAE and BUE on the Expression of Necrosis-Related Proteins In Vitro

Analogous to the results in vivo, RIP1 and RIP3 up-regulated significantly in BUE groups (*p* < 0.01). In contrast, there were no obvious changes on the levels of these proteins in EAE groups (Figure 8B).

### 2.8. The Effect of EAE and BUE on L-02 Cell Membrane

**Figure 9:**
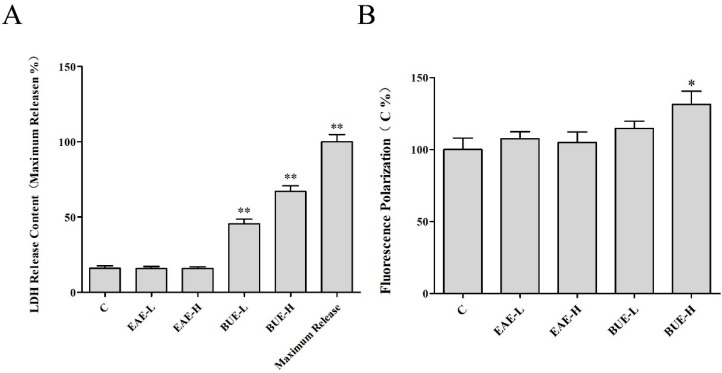
Effects of EAE and BUE (**A**) on LDH release content and (**B**) cell membrane fluidity in L-02 cells. Results are expressed as mean ± SD (*n* = 5), * *p* < 0.05, ** *p* < 0.01 compared with the control group.

Necrosis in cells can lead to membrane damage. Thus, the effects of EAE and BUE on L-02 cell membrane were evaluated. The level of lactate dehydrogenase (LDH) release is commonly considered as an indicator of cell membrane damage [12]. As shown in Figure 9A, the treatment of EAE did not induce the release of LDH. Nevertheless, the content of LDH was significantly increased in BUE groups, especially in the BUE-H group (at 67%).

Cell membrane fluidity can be detected by fluorescence polarization, by measuring the strength of the fluorescence [13]. The increase in fluorescence polarization represented the decrease in membrane fluidity. The result suggested that after 2 h treatment, a high dose of BUE could reduce membrane fluidity (*p* < 0.05), whereas EAE groups exhibited no significant effect on membrane fluidity in L-02 cells (Figure 9B).

**Figure 10:**
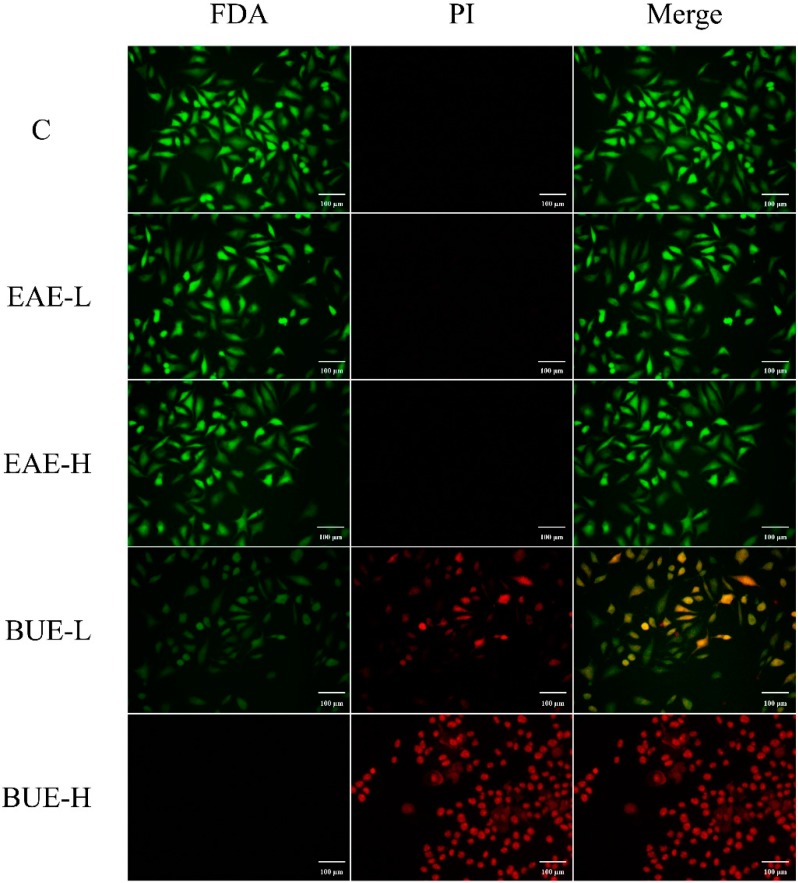
Effects of EAE and BUE on membrane permeability changes in L-02 cells. The green presented FDA^+^; the orange presented FDA^+^/PI^+^; the red presented PI^+^ (×200).

Fluorescein diacetate (FDA) and PI staining represent a classic method to detect cell membrane permeability and discriminate living cells, dead cells, and cells with membrane injury [14]. As shown in Figure 10, after 12 h treatment, only green fluorescence (FDA^+^) can be observed in the control and EAE groups, suggesting there was no obvious change in cell membrane permeability. However, in BUE groups, the red fluorescence (PI^+^) increased significantly, indicating that cell membrane injury had occurred. In the BUE-L group, both red and green fluorescence were observed with integrated cell structure, indicating cell membrane injury but that the cell was alive. In the BUE-H group, only red fluorescence was observed, indicating that the cell structure was dilapidated. The above results further confirmed that the toxicity induced by BUE was related to cell necrosis.

## 3. Discussion

Necrosis, as one process of cell death that is different from apoptosis, has attracted much attention from scholars. Necrosis exhibits morphological characteristics such as cellular organelle swelling, cell membrane rupture, intracellular content spillover, slow nuclear change, and inadequate DNA degradation [15]. The necrotic process of cell death is mainly induced by strong physical, chemical, or biological factors [16]. The irrational use of drugs can possibly induce necrosis [7], and studies have revealed that necrosis plays an important role in drug-induced liver injury [17].

The toxicity of drugs is often accompanied by weight loss [18]. After the administration of PH (5 g/kg), loss of weight and darkness of fur were clearly observed in the PH group. This dose of PH did not result in death, demonstrating that the LD_50_ of PH was greater than 5 g/kg. Histopathological evaluations were used to investigate visceral injury directly. In this research, the results exhibited prominent morphologically pathological characterizations of toxicity, especially in liver tissues. Therefore, the liver was selected as the target organ for further toxicological research.

Anti-inflammation was the traditional efficacy of *P. hookeri* and carrageenan-induced mice paw edema was the classical model for an anti-inflammatory study. The present research showed that EAE and BUE were the active fractions for anti-inflammation, which were applied for further evaluation of the liver toxicities in vivo and in vitro. Drug-induced liver injury causes abnormalities in biomarkers in serum and the morphology of tissues. ALT, AST, ALP, TBIL, and DBIL are all important biochemical indicators for the diagnosis of liver injury in clinical trials [19]. In the present study, BUE significantly increased the serum levels of ALT, AST, ALP TBIL, and DBIL, indicating that liver injury was induced by BUE. Furthermore, the hepatic histological changes substantiated the biomarker analysis.

Inflammation is often accompanied by the process of liver injury, which contains necrosis with cellular constituents being released [20]. NF-κB is a classic inflammatory signaling pathway. Normally, the NF-κB activity is regulated by binding with inhibitory units of IκB. Once stimulated, the IKK is activated and phosphorylated to degrade the IκB. NF-κB can then translocate to the nucleus and initiate gene transcription, such as some inflammatory cytokines [21]. Necrosis is considered to be un-programmed. However, a growing number of research studies have demonstrated that some types of necrosis could also be regulated by signaling molecules and that these types of necrosis could be defined as programmed necrosis. Programmed necrosis was regulated by a necrotic complex, in which RIP1 was the first identified signal molecule [22]. Successive reports showed that RIP3 was the core protein that controlled the necrosis-related signals. RIP1 and RIP3, belonging to the RIP family, owned the RIP homotypic interaction motif (RHIM), which promoted them to form necrosome [23]. Subsequently, RIP3 collected mixed lineage kinase domain-like proteins (MLKLs) to execute necrosis. Our results showed that treatment with BUE upregulated the expression of NF-κB, RIP1, and RIP3, confirming that the liver injury induced by BUE was mainly caused by necrosis.

Experiments in vitro were carried out for further evaluation of the toxicity of BUE. The main cells of the liver are hepatocytes; in addition, macrophages and endothelial cells are also distributed in the liver. Therefore, L-02, RAW264.7, and HUVEC were selected as tools to evaluate the toxicity. Cell viability results indicated that BUE was the toxic fraction, similar to the results obtained in vivo. Moreover, necrosis was further confirmed as the major factor inducing BUE toxicity by the treatment of Nec-1, the inhibitor of RIP1, which could significantly reverse the decrease in the cell viability of BUE. PI staining, a classical method to identify necrotic cells, also verified the above result. Western blot results in L-02 showed a similarity with those in liver tissue in mice. Both NF-κB signaling pathway and necrosis-related proteins RIP1 and RIP3 were upregulated by the BUE treatment.

Cell death induced by necrosis is morphologically characterized by cell and organelle swelling, which ultimately culminates in the loss of cell membrane integrity [24]. Therefore, the next research step was focused on the change of cell membrane structure. As the boundary membrane of cells, cell membrane integrity is important to maintain the normal activities of cells [25], such as material transport [26], energy conversion [27], and cell recognition [28]. When the membrane was damaged, LDH, a relatively stable cytoplasmic enzyme, was released to the outside. Its release was an important indicator reflecting the integrity of the cell membrane. Appropriate fluidity was necessary for maintaining the normal physiological function of cells. 1,6-diphenyl-1,3,5-hexatriene (DPH), a non-polar molecule, could bind to the cell membrane and emit fluorescence [29]. The increase in the fluorescence polarization indicated the decrease in the membrane fluidity. Maintaining the normal permeability of the cell membrane was crucial for the cells. FDA was transported into living cells and transformed into metabolites with green fluorescence. PI stain was embedded with DNA to emit red fluorescence. However, it could only reach the nucleus through the damaged membrane in necrotic cells since it was rejected by cells with a complete membrane. In our research, it was revealed that BUE-induced necrosis increased the release of LDH and permeability, but decreased the fluidity of the cell membrane.

## 4. Conclusions

The present study showed that n-butanol extract from *Pterocephalus hookeri* could induce liver toxicity in vivo and in vitro, which mainly induced the development of inflammation, and then led to necrosis. These results may provide a theoretical basis for the clinical rational use of this Chinese traditional herb.

## 5. Materials and Methods

### 5.1. Chemicals and Reagents

Carrageenan was acquired from Sigma-Aldrich (Shanghai, China). PI, FBS, and PBS were acquired from Servicebio (Wuhan, China). ALT, AST, ALP, TBIL, and DBIL kits were obtained from Biobase (Jinan, China). NF-κB, P-NF-κB, β-actin, and IκB antibodies were obtained from the Beyotime Institute of Biotechnology (Shanghai, China). RIP1, RIP3, and IKKα antibodies were obtained from Proteintech (Wuhan, China). 3-MA and Nec-1 were acquired from MedChem Express (Shanghai, China). LDH cytotoxicity assay kit and Z-VAD-FMK were obtained from the Beyotime Institute of Biotechnology (Shanghai, China). DPH was acquired from Aladdin (Shanghai, China). DMEM, DMSO, MTT, FDA, and PVDF membranes were obtained from Solarbio (Beijing, China). Other chemicals were of analytical grade and commercially available.

### 5.2. Plant Material and Extraction

The whole plants of *P. hookeri* were collected in Nyingchi County, Tibet Autonomous Region, China in August 2014, identified by Professor Xiaozhong Lan from the Tibet Agriculture and Animal Husbandry College. A voucher specimen (2014-CM-002) was deposited in the Herbarium of Medicinal Plants, College of Pharmaceutical Sciences, Southwest University, Chongqing, China.

Air-dried *P. hookeri* (7.2 kg) was extracted with 12 L of 95% ethanol with refluxed conditions carried out 4 times. The ethanol extract of *P. hookeri* was filtered and concentrated with reduced pressure to form a crude extract (1.9 kg). The extract was suspended in H_2_O and then completely partitioned with petroleum ether, ethyl acetate, and n-butanol, successively. The petroleum ether extract (PEE, 37 g), ethyl acetate extract (EAE, 221 g), and n-butanol extract (BUE, 560 g) were stored at 4 °C until use.

### 5.3. Animals

This research was carried out in strict accordance with the guidelines and regulations of the Ethical Committee of Southwest University of Traditional Chinese Medicine. All experimental protocols for the animal research were approved by the Institutional Animal Ethics Committee of Southwest University (Approval Number 2016-27). Date of approval: 9 September 2016. Male or female Kunming mice (20–22 g) were purchased from the Chongqing Academy of Chinese Materia Medica (Chongqing, China). All mice were kept in a room maintained with environmentally controlled conditions with suitable temperature (25 ± 1 °C), and 12 h light and 12 h dark cycles. All mice had free access to water and standard diet and were acclimatized at least one week before the experiments started.

### 5.4. Investigation of Body Weight and Pathological Section of Visceral Tissues in Mice

After exposure to an appropriate environment for seven days, the mice were randomly assigned into two groups: the control group and the PH group. We set the normal mice as the control (C) group. The PH group was intragastrically administered PH (5.0 g/kg) for 16 days, whereas the control group was treated with the same volume of 0.5% sodium carboxymethylcellulose (CMC-Na), and body weights were documented at a fixed time every three days. After the mice were sacrificed by cervical dislocation, heart, liver, spleen, lung, and kidney samples from the two groups were fixed in 4% formaldehyde, dehydrated, embedded in paraffin, cut into 5–7 µm sections, and stained with H&E stain. The histological analysis was observed using an optical microscope (Olympus, Tokyo, Japan). Photographs of each slide were observed with 200× magnification vision.

### 5.5. Investigation of Liver Toxicity on EAE and BUE In Vivo

Carrageenan-induced mice paw edema was tested using paw volume as inflammatory parameters [5]. The method of anti-inflammatory testing is shown in the Supplementary Materials. After serum was collected for liver biochemical indicator detection (BK-400 automatic biochemistry analyzer, Biobase, Jinan, China), mice were sacrificed and liver tissue was obtained for the H&E stain with a subsequent histological assessment. The histological analysis was the same as the former procedure.

### 5.6. Cell Culture and Treatment

L-02, RAW264.7, and HUVEC cell lines were obtained from the Shanghai Institute of Cell Biology, Chinese Academy of Sciences (Shanghai, China). Three of them were cultured in DMEM supplemented with 10% FBS, 100 U/mL penicillin and streptomycin. All cells were cultured at 37 °C in an incubator containing 5% CO_2_ and the culture medium was replenished every 2–3 days. We set the normal cells as the control (C) group. Three cells in the EAE and BUE groups were treated for 24 h with gradient concentrations (10, 20, 40, 80, 160 μg/mL) of them dissolved in DMSO. Three cells in Z-VAD-FMK, 3-MA, and Nec-1 were treated for 24 h with concentrations of 20 μmol/L, 5 mmol/L, and 50 μmol/L, respectively. The L-dose and H-dose in EAE and BUE groups were 80 and 160 μg/mL.

### 5.7. Cell Viability Assay

The viability of L-02, RAW264.7, and HUVEC cells was tested according to the MTT instructions. Briefly, approximately 5000 cells per well were seeded into 96-well plates containing complete DMEM (100 μL) and continuously cultured for 24 h in an incubator. Following the various treatments, each well was supplemented with 10 μL of MTT (5 mg/mL) solution and incubated at 37 °C for another 4 h. The absorbance at 490 nm was assessed by a multifunctional microplate reader (Gene Company, Hong Kong, China).

### 5.8. PI Staining Assay

L-02 cells were cultured at the density of 1 × 10^5^ cells/well in 24-well cell culture plates and then treated with EAE and BUE in concentrations of 80 and 160 μg/mL. After treatment for 24 h, cells were washed twice with PBS and DMEM (450 μL) was added into each well. Then, a 50 μL portion of PI (100 μg/mL) staining was added for 30 min and observed under a fluorescent microscope. Photographs of each slide were observed with 200× magnification vision.

### 5.9. LDH Leakage Assay

L-02 cells were seeded into 96-well plates at a density of 5000 cells per well. After 24 h for adherence, cells were treated with EAE and BUE in concentrations of 80 and 160 μg/mL for 24 h. LDH release rates were detected using the LDH cytotoxicity assay kit after drug treatment. The LDH assay was estimated by using the LDH detection kit according to the manufacturer’s instructions and measured at a wavelength of 490 nm with a multifunctional microplate reader.

### 5.10. L-02 Cell Membrane Fluidity Assessment

L-02 cells were seeded into 6-well plates with a cell density of 1 × 10^6^ per well. After 24 h for adherence, cells were treated with EAE and BUE in concentrations of 80 and 160 μg/mL for 2 h. Trypsin was applied to digest the cells. The suspended cells were transferred to the 1.5 mL centrifuge tube, centrifuged at 1000 rpm/min at room temperature for 5 min to collect the cells. After washing twice with PBS, cells were loaded with 1 mL DPH fluorescent probe (2 μmol/L), mixed fully, and incubated at 37 °C for 20 min. Finally, we inoculated the cell suspension into black 96-well plates with 100 μL per hole. Fluorescence polarization measurements with the parameter of 362 nm excitation and 432 nm emission were carried out on a multifunctional microplate reader.

### 5.11. L-02 Cell Membrane Permeability Assessment

L-02 cells were seeded into 24-well plates with a cell density of 1 × 10^5^ per well. After 24 h of adherence, cells were treated with EAE and BUE in concentrations of 80 and 160 μg/mL for 12 h, respectively. After washing twice with PBS, DMEM (450 μL) was added into each well. Then, a 50 μL portion of PI (100 μg/mL) staining was added for 30 min and then a 2 μL portion of FDA (5 mg/mL) was added for 10 min at 37 °C. At the end of staining, cells were cleaned twice by PBS, and then observed under a fluorescent microscope. Photographs of each slide were observed with 200× magnification vision.

### 5.12. Western Blot Analysis

Liver tissue and L-02 cell lysates for EAE or BUE treatment were prepared on ice using RIPA buffer containing phosphatase and protease inhibitors and centrifuged at 10,000 rpm for 15 min at 4 °C. Protein concentration of each group was measured by a BCA protein assay kit (Beyotime Institute of Biotechnology, Shanghai, China). All samples were electrophoresed and segregated using SDS–PAGE and transferred to PVDF membranes. Then, the PVDF membranes were blocked with PBS buffer containing 5% skimmed milk and incubated with anti-P-NF-κB, anti-NF-κB, anti-IκB, anti-IKKα, anti-RIP1, anti-RIP3, and anti-β-actin at a dilution of 1:1000 overnight at 4 °C. Horseradish peroxidase-conjugated anti-mouse/rabbit IgG was used as secondary antibody at a dilution of 1:2000 and subsequently visualized by an imaging system (Tanon 5200, Shanghai, China).

### 5.13. Statistical Analysis

Statistical analysis was performed by GraphPad Prism version 5.0 software and the results were expressed as mean ± SD. A one-way analysis of variance (ANOVA) followed by Dunnett’s *t*-test was used to assess the statistical significance between the groups, and statistical significance was defined as *p* < 0.05 (* or #) and *p* < 0.01 (** or ##).

## Supplementary Materials

The following are available online at https://www.mdpi.com/2072-6651/11/3/142/s1, Anti-inflammatory test of PEE, EAE, and BUE against carrageenan-induced paw edema in mice.
