# Supplementary material for: Evaluation of the Liver Toxicity of Pterocephalus hookeri Extract via Triggering Necrosis
Source: Toxins (Basel). 2019 Mar 2;11(3):142. doi: 10.3390/toxins11030142 (PMC6468695; doi:10.3390/toxins11030142)
Supplement: Supplementary file 1 [file toxins-11-00142-s001.pdf]

Supplementary Materials: Evaluation of the Liver Toxicity of *Pterocephalus hookeri* Extract via Triggering Necrosis

Rui Wang, Zhaoyue Dong, Xiaolong Zhang, Jingxin Mao, Fancheng Meng, Xiaozhong Lan, Zhihua Liao and Min Chen

Anti-Inflammatory Test of PEE, EAE and BUE against Carrageenan-Induced Paw Edema in Mice

All mice were divided into 11 groups including control group, Mod group, PEE-L group, PEE-M group, PEE-H group, EAE-L group, EAE-M group, EAE-H group, BUE-L group, BUE-M group and BUE-H group. We set the normal mice as the control (C) group. The low (L), middle (M) and high (H) dose of PEE, EAE and BUE (200, 400, 800 mg/kg) were intragastric administered to mice per day for 7 days, while the control group and Mod group were treated with the same volume of 0.5% CMC-Na. At 8th day, 50 μL carrageenan was injected intradermal into the plantar surface of the right hind paw of each mouse except the control group. The different dose of PEE, EAE and BUE were intragastric administered 1 h prior to carrageenan injection. The paw volume was determined at 0.5 h, 1 h, 2 h, 4 h, and 8 h after carrageenan injection by PV-200 plethysmometer (Taimeng, Chengdu, China). After serum collected, all animals were sacrificed to obtain the liver tissues.

Table S1. Paw volume parameters in mice (mL) with treatment of PEE, EAE and BUE.

Group	Pre-inject	0.5 h	1 h	2 h	4 h	8 h
C	0.18 ± 0.02	0.22 ± 0.02	0.25 ± 0.02	0.24 ± 0.03	0.27 ± 0.02	0.23 ± 0.03
Mod	0.15 ± 0.01	0.30 ± 0.03$^{##}$	0.35 ± 0.05$^{##}$	0.34 ± 0.05$^{##}$	0.47 ± 0.04$^{##}$	0.37 ± 0.06$^{##}$
PEE-L	0.16 ± 0.03	0.32 ± 0.03	0.33 ± 0.04	0.32 ± 0.05	0.46 ± 0.05	0.38 ± 0.04
PEE-M	0.16 ± 0.03	0.32 ± 0.05	0.34 ± 0.03	0.33 ± 0.03	0.46 ± 0.05	0.40 ± 0.05
PEE-H	0.16 ± 0.02	0.30 ± 0.03	0.33 ± 0.03	0.33 ± 0.02	0.45 ± 0.05	0.38 ± 0.08
EAE-L	0.17 ± 0.02	0.31 ± 0.05	0.35 ± 0.05	0.33 ± 0.04	0.41 ± 0.05*	0.37 ± 0.03
EAE-M	0.14 ± 0.02	0.30 ± 0.03	0.34 ± 0.02	0.28 ± 0.05*	0.32 ± 0.03**	0.34 ± 0.03
EAE-H	0.16 ± 0.02	0.31 ± 0.02	0.29 ± 0.03**	0.25 ± 0.03**	0.32 ± 0.02**	0.31 ± 0.03*
BUE-L	0.15 ± 0.01	0.29 ± 0.03	0.35 ± 0.03	0.36 ± 0.04	0.43 ± 0.03	0.35 ± 0.05
BUE-M	0.15 ± 0.01	0.34 ± 0.04	0.32 ± 0.03	0.31 ± 0.04	0.40 ± 0.03**	0.31 ± 0.05
BUE-H	0.16 ± 0.01	0.32 ± 0.05	0.34 ± 0.02	0.32 ± 0.03	0.38 ± 0.03**	0.30 ± 0.05*

Values expressed as mean ± SD (n = 8), # $p < 0.05$, ## $p < 0.01$ compared with the control group; * $p < 0.05$, ** $p < 0.01$ compared with the Mod group.
